# A Diagnostic Delay: Respiratory Muscle Weakness in Dermatomyositis Masquerading as Pneumonia

**DOI:** 10.7759/cureus.100112

**Published:** 2025-12-26

**Authors:** Mansi Jain, Vijay Kumar Doddapaneni, Bakht Rahman, Nadia Aslam

**Affiliations:** 1 Internal Medicine, St. Vincent Medical Center, Toledo, USA; 2 Internal Medicine, Community Regional Medical Center, Fresno, USA

**Keywords:** dermatomyositis, gottron's papules, interstitial lung disease, pneumonia, respiratory muscle weakness

## Abstract

Dermatomyositis is an idiopathic inflammatory myopathy with characteristic involvement of skin and muscle. While interstitial lung disease (ILD) is a well-recognized pulmonary manifestation, hypoventilation due to respiratory muscle weakness remains underrecognized and may masquerade as primary lung pathology. We present the case of a 40-year-old woman with a history of polycystic ovarian syndrome who initially presented with dyspnea and was diagnosed with presumed pneumonia based on computed tomography findings. Despite antibiotic therapy, her symptoms progressively worsened over the next three weeks, during which evolving proximal muscle weakness and a diffuse pruritic rash developed, yet the diagnosis remained elusive. Repeat imaging ultimately suggested atelectasis rather than pneumonia; infectious workup was negative, and marked elevation of muscle enzymes with characteristic cutaneous findings, including the V sign and Gottron’s papules, raised suspicion for dermatomyositis, later confirmed by biopsy. With high-resolution computed tomography of the chest showing no ILD and cardiopulmonary evaluation otherwise unremarkable, her dyspnea was attributed to extrapulmonary restrictive disease secondary to respiratory muscle weakness. She showed significant clinical improvement following immunosuppressive therapy.

This case underscores how respiratory muscle weakness in dermatomyositis can mimic primary pulmonary disease and contribute to diagnostic delay. Recognizing evolving weakness and pathognomonic rashes, even in the absence of ILD, can shorten time to diagnosis and improve outcomes.

## Introduction

Dermatomyositis is an autoimmune inflammatory myopathy characterized by cutaneous manifestations and proximal muscle weakness. Pulmonary involvement is a significant source of morbidity and mortality in these patients, with interstitial lung disease (ILD) being the most widely recognized [1]. However, respiratory muscle weakness, particularly in the absence of overt parenchymal lung disease, is a lesser-known but clinically significant manifestation [2]. This case highlights a diagnostic challenge in which a patient with dermatomyositis presented primarily with progressive dyspnea and was initially misdiagnosed with pneumonia, delaying appropriate treatment.

## Case presentation

**Figure 1:**
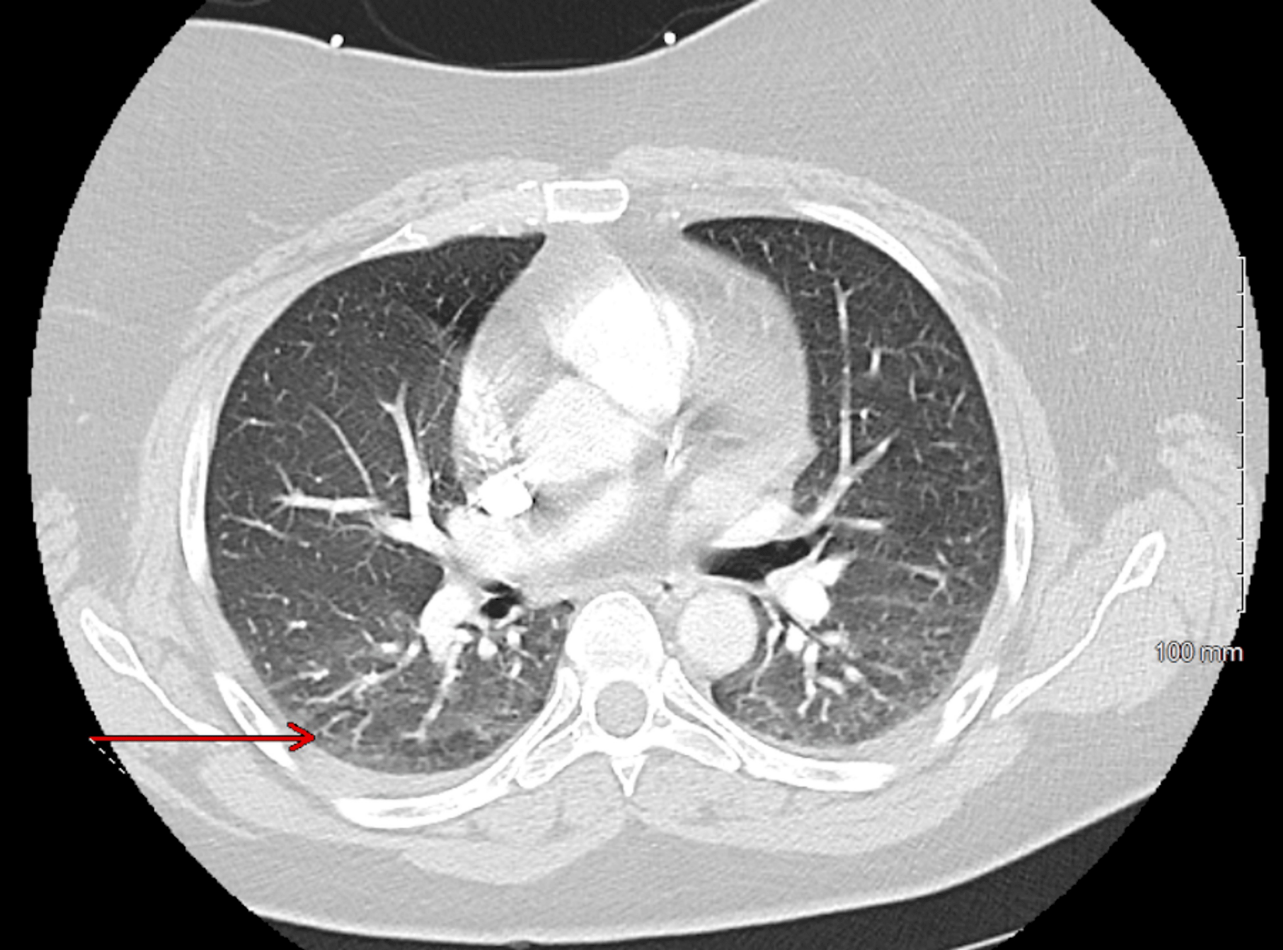
CTPE from the first ED visit showing bilateral ground-glass infiltrates and trace pleural effusions, as indicated by the red arrow. CTPE: Computed tomography pulmonary embolism

The patient is a 40-year-old woman with a past medical history of polycystic ovarian syndrome, who initially presented to the emergency department (ED) with dyspnea on exertion without any associated symptoms. At that time, complete blood count and basic metabolic panel were unremarkable. Influenza A and B were negative. D-dimer was 0.70 µg/mL (reference range: < 0.5 µg/mL). A computed tomography pulmonary embolism (CTPE) scan showed bilateral ground-glass infiltrates with trace pleural effusions, suggestive of multifocal pneumonia versus pulmonary edema (Figure 1). She was administered a single dose of ceftriaxone and discharged on a course of azithromycin.

Three weeks later, she presented with worsening shortness of breath, increasing myalgias, and interval development of a diffuse pruritic erythematous maculopapular rash. She also reported difficulty performing activities of daily living, including dressing, combing her hair, and bathing. She denied fever, chills, headache, dizziness, blurry vision, cough, dysphagia, chest pain, abdominal pain, nausea, vomiting, dysuria, diarrhea, or constipation as well as any recent use of new medications, including over-the-counter drugs or supplements.

**Figure 2:**
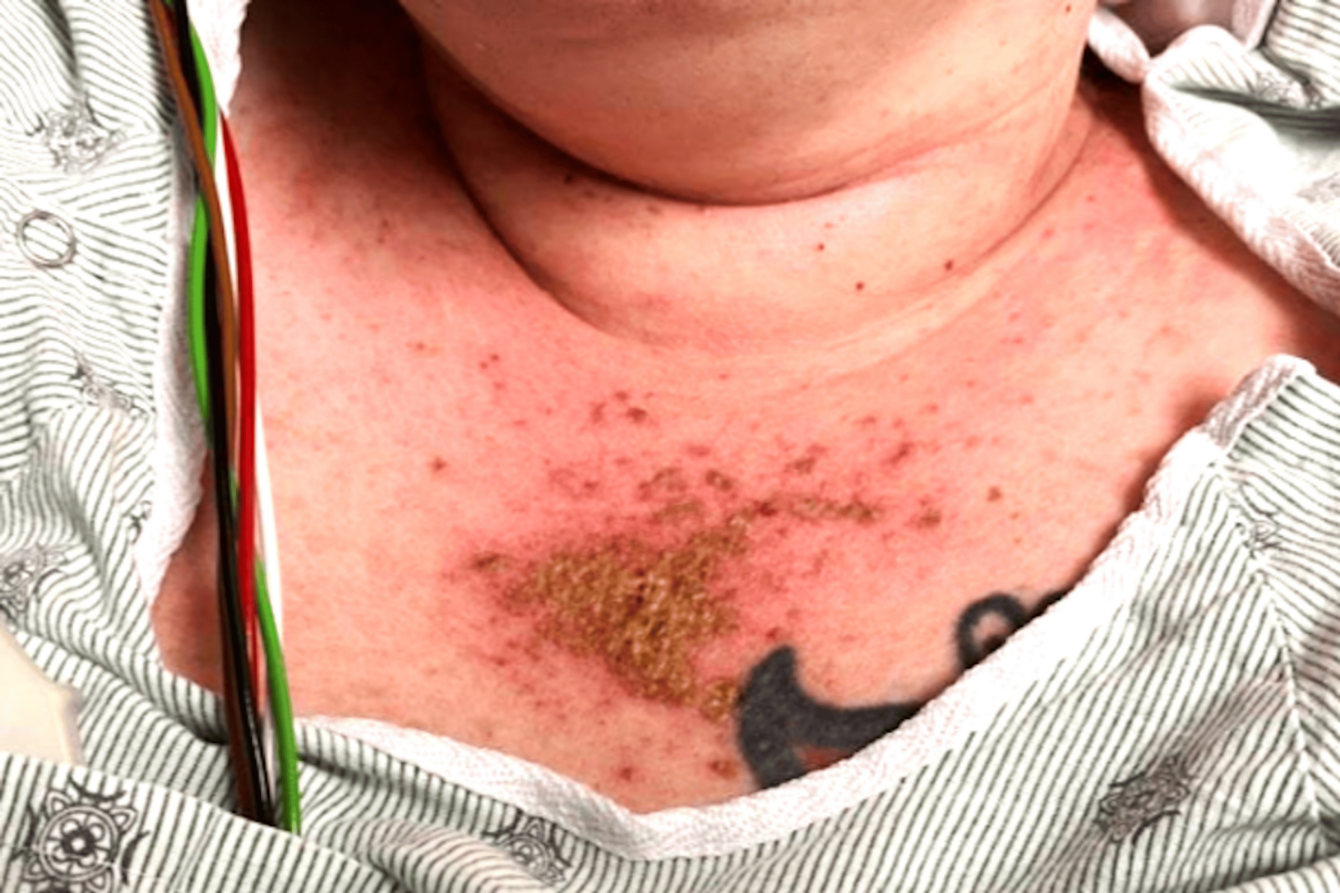
Image of the anterior lower part of the neck and chest wall in the sun-exposed area with hyperpigmented reddish maculopapular rash with scaling

**Figure 3:**
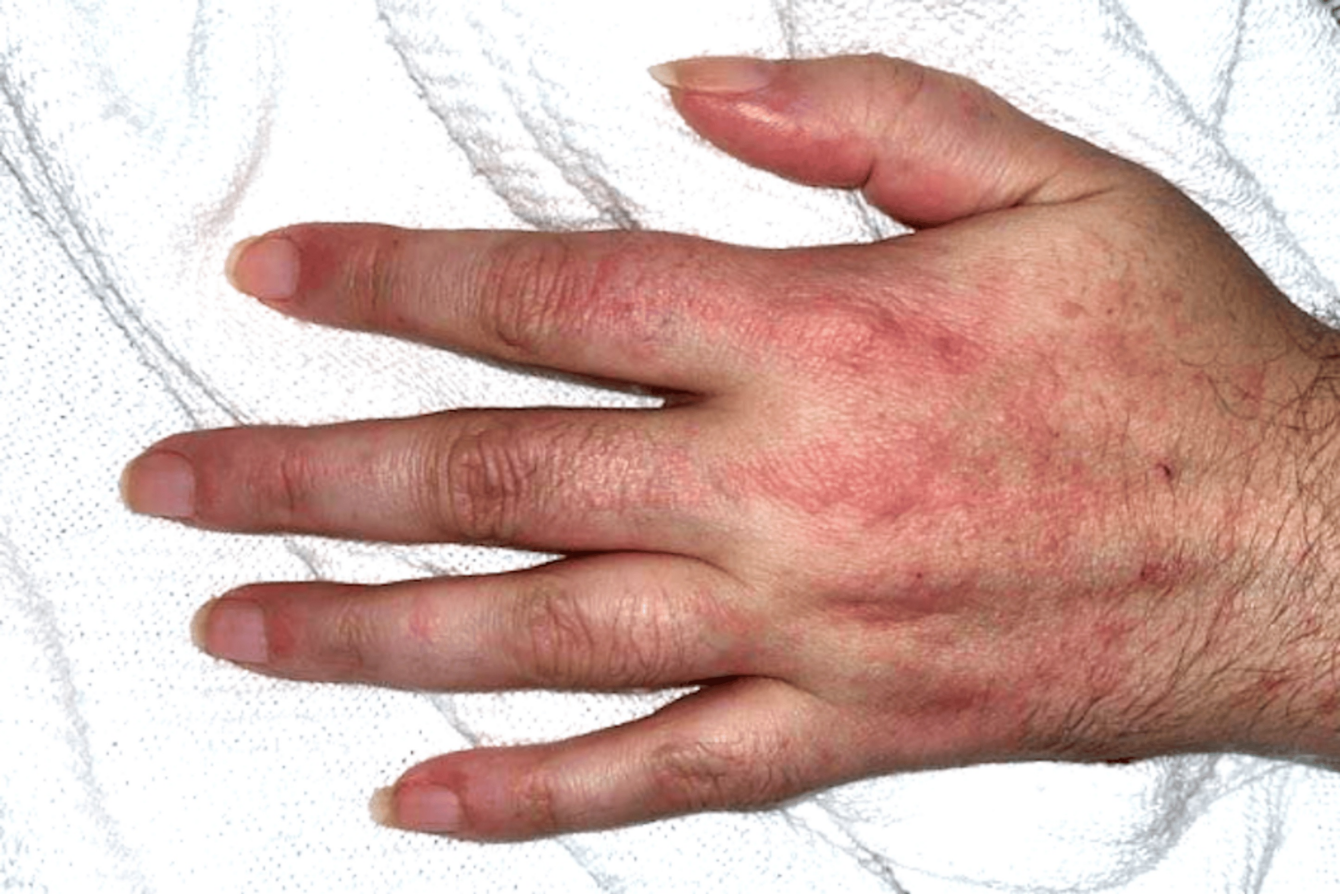
Image of the left hand showing multiple hyperkeratotic flat papules on the dorsum of the metacarpophalangeal and the dorsum of the fingers.

Vital signs on admission were blood pressure 121/86 mmHg, heart rate 105 bpm, temperature 97.2°F, respiratory rate 18 breaths per minute, and oxygen saturation was within normal limits on room air. Physical examination revealed a diffuse pruritic maculopapular rash involving the neck, upper chest (Figure 2), hands (Figure 3), lower back, and upper thighs, with no mucosal involvement. Auscultation of the lungs revealed clear breath sounds. Proximal muscle strength was diminished in both upper and lower extremities, with muscle power graded as 3/5.

Laboratory results on admission are shown as follows (Table 1). Her initial laboratory evaluation was notable for hyponatremia, and in the context of low urine sodium, high urine osmolality, and clinical evidence of dehydration, the findings were most consistent with hypovolemic hyponatremia, which was corrected with appropriate fluid resuscitation.

**Table 1:** Laboratory results on admission

Test	Reference Range	Result
Basic Metabolic Panel		
Sodium	135–144 mmol/L	123
Potassium	3.7–5.3 mmol/L	4.7
Chloride	98–107 mmol/L	92
Carbon dioxide	20–31 mmol/L	18
Blood urea nitrogen	6–20 mg/dL	29
Creatinine	0.5–0.9 mg/dL	0.6
Anion gap	9–17 mmol/L	13
Estimated glomerular filtration rate	>60 mL/min/1.73 m²	>60
Renal and Electrolyte Studies		
Serum osmolality	275–295 mOsm/kg	285
Urine osmolality	300–900 mOsm/kg*	208
Urine sodium	Variable	20
Complete Blood Count		
White blood cell count	3.5–11.3 ×10³/µL	11.2
Red blood cell count	3.95–5.11 ×10⁶/µL	5.34
Hemoglobin	11.9–15.1 g/dL	16.1
Hematocrit	36.3–47.1%	47.7
Mean corpuscular volume	82.6–102.9 fL	89.3
Mean corpuscular hemoglobin	25.2–33.5 pg	30.1
Mean corpuscular hemoglobin concentration	28.4–34.8 g/dL	33.8
Red cell distribution width	11.8–14.4%	14.0
Platelet count	138–453 ×10³/µL	257
Mean platelet volume	8.1–13.5 fL	10.9
Inflammatory Markers		
C-reactive protein	0.0–5.0 mg/L	5.1
Erythrocyte sedimentation rate	0–20 mm/hr	12
Liver Function Tests		
Albumin	3.5–5.2 g/dL	2.3
Total protein	6.4–8.3 g/dL	4.8
Albumin-to-globulin ratio	1.0–2.5	0.9
Alkaline phosphatase	35–104 U/L	96
Alanine aminotransferase	5–33 U/L	226
Aspartate aminotransferase	<32 U/L	568
Total bilirubin	0.3–1.2 mg/dL	0.2
Direct bilirubin	<0.3 mg/dL	0.1
Indirect bilirubin	0.0–1.0 mg/dL	0.1
Endocrinology Labs		
Thyroid-stimulating hormone	0.27–4.2 µIU/mL	4
Free thyroxine	0.9–1.7 ng/dL	1.1
Respiratory Viral Polymerase Chain Reaction (PCR) Panel		
Adenovirus PCR	Not detected	Not detected
Bordetella pertussis PCR	Not detected	Not detected
Chlamydia pneumoniae PCR	Not detected	Not detected
Human metapneumovirus PCR	Not detected	Not detected
Influenza A and B PCR	Not detected	Not detected
Parainfluenza virus types 1–4 PCR	Not detected	Not detected
Respiratory syncytial virus PCR	Not detected	Not detected
Rhinovirus/Enterovirus PCR	Not detected	Not detected
Mycoplasma pneumoniae PCR	Not detected	Not detected
Bordetella parapertussis PCR	Not detected	Not detected
SARS-CoV-2 PCR	Not detected	Not detected
Microbiology		
Blood culture	—	No growth
Urine culture	—	No growth

**Figure 4:**
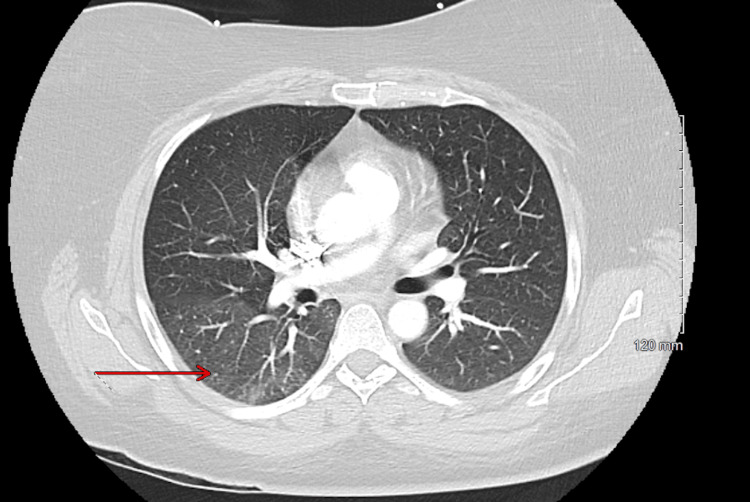
CTPE from the second ED visit demonstrating persistent mild bilateral infiltrates suggestive of atelectasis or pneumonitis, as indicated by the red arrow. CTPE: Computed tomography pulmonary embolism

Chest X-ray was unremarkable. Repeat CTPE (Figure 4) showed persistent mild bilateral infiltrates suggestive of atelectasis or pneumonitis.

Given her worsening symptoms, she was started on levofloxacin for broader antimicrobial coverage and was prescribed topical corticosteroids to manage the inflammatory rash. A comprehensive hepatic workup was initiated in light of elevated liver enzymes. The patient reported occasional alcohol use. Viral hepatitis panel (hepatitis A, B, C), Epstein-Barr virus, cytomegalovirus, ceruloplasmin, alpha-1 antitrypsin, antimitochondrial antibodies, ferritin, gamma-glutamyl transferase, antinuclear antibody, and antineutrophil cytoplasmic antibodies were all negative. Liver ultrasound was unremarkable. Gastroenterology concluded that the transaminitis was likely of extrahepatic origin.

Creatine kinase (CK) was markedly elevated at 29,760 U/L (reference range: 26-192 U/L), and serum myoglobin was 6,550 ng/mL (reference range: 25-58 ng/mL). She denied statin use. The presence of proximal muscle weakness, elevated muscle enzymes, and characteristic rashes, such as the V-shawl sign (Figure 2) and Gottron’s papules (Figure 3), raised strong suspicion for dermatomyositis. Rheumatology was consulted, and skin and muscle biopsies were performed.

Skin biopsy from the thigh showed interface dermatitis with subepidermal vesiculation. Muscle biopsy of the proximal thigh demonstrated findings consistent with dermatomyositis.

**Figure 5:**
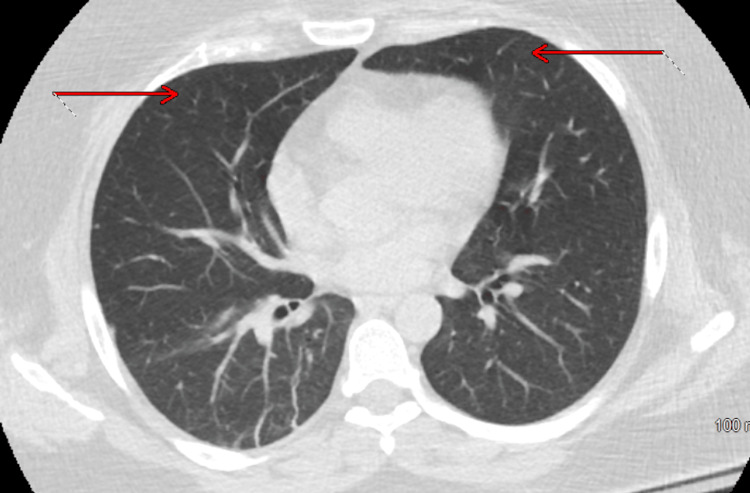
HRCT showing the absence of parenchymal lung disease, as indicated by the red arrows. HRCT: High-resolution computed tomography

High-resolution computed tomography (HRCT) of the chest showed no evidence of parenchymal lung disease (Figure 5). The earlier CT abnormalities were likely attributable to dependent atelectasis, as the HRCT performed in the prone position demonstrated improved aeration of the basal lung regions, a finding that helps distinguish atelectasis from pneumonia. The fluoroscopic diaphragmatic motion study (SNIFF test) was negative, and the echocardiogram (ECHO) revealed normal ejection fraction and right ventricular systolic pressure. Pulmonology concluded that her dyspnea was most consistent with extrapulmonary restrictive lung disease due to respiratory muscle weakness. A CT scan of the abdomen and pelvis was negative for occult malignancy.

The patient was started on high-dose corticosteroids, methotrexate, and intravenous immunoglobulin (IVIG) with significant clinical improvement, including a reduction in CK from 22,950 to 5,081 U/L (reference range: 26-192 U/L). She was discharged to an acute rehabilitation facility on prednisone 40 mg twice daily and methotrexate 15 mg weekly with outpatient rheumatology follow-up and a recommendation for pulmonary function testing (PFT) and a sleep study, which unfortunately she did not complete.

## Discussion

Dermatomyositis is an idiopathic autoimmune inflammatory myopathy characterized by proximal muscle weakness and hallmark cutaneous manifestations. The incidence is approximately 1 per 100,000, with a prevalence of 13 per 100,000, and it is more common in women [3,4]. Pulmonary complications account for a substantial proportion of morbidity and mortality in these patients [1]. While ILD is the most widely recognized pulmonary manifestation, other mechanisms such as aspiration from oropharyngeal dysfunction and hypoventilation due to respiratory muscle weakness can also contribute to respiratory symptoms, even when parenchymal lung disease is absent [5]. Supporting this concept, Selva O’Callaghan et al. reported pulmonary involvement in 50 of 81 patients with polymyositis or dermatomyositis, including 18 who demonstrated a restrictive pattern attributable to respiratory muscle weakness rather than ILD, underscoring that dyspnea in inflammatory myopathies may arise from impaired respiratory muscle function despite unremarkable imaging [6].

Our case reflects this diagnostic complexity. The patient’s dyspnea on exertion was initially attributed to presumed pneumonia, yet her symptoms did not improve with antibiotic therapy. The progression of muscle weakness, the appearance of characteristic rash, the negative infectious evaluation, the elevated muscle enzymes and the absence of parenchymal abnormalities on HRCT shifted the diagnostic focus toward respiratory muscle dysfunction. Her marked and rapid clinical improvement following initiation of immunosuppressive therapy further supports this mechanism.

Published literature reinforces the importance of recognizing respiratory muscle involvement in inflammatory myopathies. Braun et al. demonstrated reductions in maximal inspiratory and expiratory pressures among patients with proximal myopathies, illustrating how weakened respiratory musculature can produce a restrictive ventilatory pattern even when imaging appears normal [7]. Additional reports describe severe respiratory compromise arising from muscle involvement rather than intrinsic lung disease. Selva O’Callaghan et al. identified cases of chronic hypercapnic respiratory failure requiring long-term ventilatory support, and Chong et al. documented diaphragmatic paralysis leading to cardiac arrest, with improvement following immunosuppressive therapy in both reports [8,9]. Ali et al. similarly described hypercapnic respiratory failure during pregnancy in polymyositis, despite normal pulmonary imaging, highlighting that profound ventilatory impairment can result solely from respiratory muscle dysfunction [10].

Collectively, these findings expand the understanding of pulmonary involvement in inflammatory myopathies beyond ILD. Respiratory muscle weakness can present subtly yet produce significant ventilatory impairment, even when routine imaging and cardiopulmonary evaluations are unrevealing. In such situations, clinical judgment guided by proximal muscle weakness, characteristic cutaneous findings, and supporting laboratory or biopsy results becomes essential. Early recognition and timely initiation of immunosuppressive therapy are crucial to prevent progression to ventilatory failure and to improve patient outcomes.

Several limitations in the evaluation of this case warrant consideration, many of which reflect the practical constraints and acuity of inpatient care where certain diagnostic studies cannot always be completed. PFT, including measurements of inspiratory and expiratory pressures and positional changes in forced vital capacity, was not completed, limiting objective confirmation of respiratory muscle weakness. Myositis-specific antibody testing, which offers important prognostic and phenotypic information and helps define the risk of interstitial lung disease, was not available at the time of assessment. Electromyography and magnetic resonance imaging of the proximal muscles were also not performed. Although the muscle biopsy provided definitive evidence of dermatomyositis, these additional modalities could have further clarified the extent and distribution of muscle involvement. Despite these limitations, the characteristic cutaneous findings, profound elevation in muscle enzymes, definitive biopsy results, absence of parenchymal involvement on HRCT, and the patient’s substantial improvement with immunosuppressive therapy strongly support the conclusions drawn from this case.

## Conclusions

This case underscores the importance of recognizing respiratory muscle weakness as a potentially serious yet treatable pulmonary manifestation of dermatomyositis. In patients presenting with unexplained dyspnea, nonspecific imaging findings, and progressive proximal muscle weakness, clinicians should maintain a high index of suspicion for myositis-related respiratory involvement. Prompt recognition and initiation of immunosuppressive therapy can significantly improve outcomes and reduce the risk of long-term ventilatory failure.
